# Epigenetics in SLE

**DOI:** 10.1007/s11926-017-0685-1

**Published:** 2017-07-27

**Authors:** Christian Michael Hedrich

**Affiliations:** 1 Division of Paediatric Rheumatology and Immunology, Children’s Hospital Dresden, Faculty of Medicine Carl Gustav Carus, TU Dresden, Dresden, Germany; 2 Department of Women᾿s & Children᾿s Health, Institute of Translational Medicine, University of Liverpool, Liverpool, UK; 3 Department of Paediatric Rheumatology, Alder Hey Children᾿s NHS Foundation Trust Hospital, East Prescott Road, Liverpool, L14 5AB UK

**Keywords:** Lupus, Epigenetic, Inflammation, Methylation, Hydroxymethylation, Histone, Non-coding RNA

## Abstract

**Purpose of Review:**

Systemic lupus erythematosus is a severe autoimmune/inflammatory condition of unknown pathophysiology. Though genetic predisposition is essential for disease expression, risk alleles in single genes are usually insufficient to confer disease. Epigenetic dysregulation has been suggested as the missing link between genetic risk and the development of clinically evident disease.

**Recent Findings:**

Over the past decade, epigenetic events moved into the focus of research targeting the molecular pathophysiology of SLE. Epigenetic alteration can be the net result of preceding infections, medication, diet, and/or other environmental influences. While altered DNA methylation and histone modifications had already been established as pathomechanisms, DNA hydroxymethylation was more recently identified as an activating epigenetic mark.

**Summary:**

Defective epigenetic control contributes to uncontrolled cytokine and co-receptor expression, resulting in immune activation and tissue damage in SLE. Epigenetic alterations promise potential as disease biomarkers and/or future therapeutic targets in SLE and other autoimmune/inflammatory conditions.

## Introduction

Systemic lupus erythematosus (SLE) is a severe autoimmune/inflammatory condition that can affect any organ of the human body [1]. Systemic inflammation and tissue damage contribute to the clinical picture of SLE and can cause severe sequelae that may result in disability or death. The pathophysiology of SLE is complex. While gain- or loss-of-function mutations in single genes may result in SLE or an SLE-like picture in a small subset of patients (approximately 1–4%), most SLE patients suffer from more pathophysiologically complex forms that remain incompletely understood [1]. Most patients are genetically predisposed to the development of SLE. However, so-called risk alleles are by themselves not strong enough to confer “full-blown” disease. In such cases, additional factors, including female gender and hormonal factors, environmental triggers (including infections, medication, exposure to toxins, and chemicals), immune regulatory factors, and epigenetic events provide additional pathophysiological impact that contributes to disease expression.

Epigenetic mechanisms are reversible as well as heritable events that govern gene expression without altering the underlying DNA sequence. They control the accessibility of DNA to the transcriptional complex, including transcription factors and RNA polymerases. Thus, epigenetic events control gene expression in a tissue- and signal-specific manner. Epigenetic events are responsible for the fact that (with the exception of gametes that carry only half of the genetic information) all cells of the human body carry the same genetic information, while exhibiting variable and sometimes highly specialized phenotypes (e.g., liver cells vs. adipose tissue vs. lymphocytes).

A number of molecular mechanisms contribute to what is called “the epigenome,” including DNA methylation, histone modifications, and non-coding transcripts. Alterations to the epigenome are involved in the dysregulation of signaling molecules and receptors in various autoimmune/inflammatory conditions, including SLE [2–6]. Thus, epigenetic events are interesting targets in the search for disease pathomechanisms, and even promise the potential of future therapeutic interventions. Though not “officially labelled” as epigenetic treatments, several medications are currently being used to modify epigenetic marks, thereby providing further evidence for a central involvement of the epigenome in immune regulation and disease pathology [1–6].

## DNA Methylation

DNA methylation is probably the most well-studied epigenetic event. Adding a methyl-group to the 5′ carbon position of cytosine in cytosine-phosphate-guanosine (CpG) dinucleotides is a potent epigenetic mechanism. It controls the accessibility of regulatory regions to transcription factors, transcriptional co-activators, and RNA polymerases. The central involvement of DNA methylation in the pathophysiology of SLE was further underscored when Javierre et al. [7] demonstrated significantly variable DNA methylation patterns in disease discordant monozygotic twins. Indeed, in genetically identical twins, altered DNA methylation patterns differentiate immune cells from SLE patients and those of healthy siblings [7].

DNA methylation is conferred by DNA methyltransferase (DNMT) enzymes. Historically, two classes of DNMTs were distinguished: (i) maintenance DNMTs (DNMT1) were believed solely responsible for re-methylation during cell division, while (ii) de novo DNMTs (DNMT3a and 3b) were claimed to confer DNA methylation independent of pre-existing patterns [2–4]. More recently, it has become increasingly clear that the historic classification was an over-simplification and that maintenance DNMTs can also confer de novo DNA methylation [3]. Indeed, DNA methylation and its regulation are more complex than previously assumed. Multiple proteins are involved in its regulation, and dysregulation of some may contribute to inflammation. Methyl-CpG-binding proteins are responsible for the solidification of transcriptional repression. Six family members were reported, including methyl-CpG-binding domain (MBD)1 through MBD4, Kaiso, and methyl-CpG-binding protein (MeCP)2 [8, 9]. Methylated-CpG-binding proteins are structural proteins that recruit histone deacetylases (HDACs) and other chromatin remodeling factors. MBD proteins thereby aid in translating DNA methylation into histone modifications (see below) [3].

Aberrant DNA methylation was first linked to altered gene expression in cancer [10, 11]. More recently, disrupted DNA methylation patterns were established as a central contributor to autoimmune/inflammatory disorders, including SLE [6]. DNA methylation patterns are complex with areas of increased DNA methylation and areas with reduced DNA methylation co-existing in cells or tissues of individuals with autoimmune/inflammatory conditions [2–6] (Table 1).

**Table 1:** Altered DNA methylation in SLE

Gene	Cellular sources	Cell type studied	Function	Effects in SLE	Ref.
Reduced DNA methylation					
*CD6* (cluster of differentiation 6)	On the cell surface of T cells and some other immune cells	CD3^+^ T cells	Important for continuation of T cell activation	Enhanced T cell activation	[12]
*CREM* (cAMP response element modulator) promoter P1	T cells, multiple other cells and tissues	CD3^+^ T cells, CD4^+^ T cells, effector CD4^+^ T cells	Transcription factor, regulating multiple cellular functions, including cytokine expression in T cells	Involved in the generation of DN T cells, promotes effector phenotypes through the regulation of IL-2 and IL-17 in CD4^+^ T cells	[13, 14•, 15–19]
*ESR1* (estrogen receptor 1 or α)	Ubiquitously expressed	PBMCs	Nuclear receptor activated by estrogen	Increased estrogen signaling, contributing to immune activation (through CREMα?)	[20, 21]
Human endogenous retroviral elements (HERVs)	Generally all human cells, in SLE: B and T cells	CD4^+^ T cells, CD8^+^ T cells, B cells	None. HERVs are remainders of ancient retroviral infections and usually silenced by epigenetic events	Increased expression of interferon-related genes raised the possibility of a viral contribution to SLE; HERV protein products have been demonstrated to induce (auto-) antibody production	[22–25]
*IFI44L* (interferon-induced 44-like protein)	Immune cells	PBMCs	While the function of IFI44L is unknown, increased IFI44L expression is a component of the type 1 interferon response signature and part of the cellular response to viral infection	“Interferon signature” gene; global immune activation	[26•, 27, 28]
*IKZF4* (IKAROS family zinc finger 4, encoding for Eos)	Lymphocytes	CD4^+^ T cells	Member of the IKAROS family of transcription factors, implicated in the control of lymphoid development	“Interferon signature” gene; global immune activation	[29]
*IL4*	CD4^+^ T cells (mainly Th2), NK cells	CD4^+^ T cells	Differentiation of Th2 cells, maturation, and reduced apoptosis, B cell maturation, immunoglobulin class switch (IgE), eosinophil migration, reduced apoptosis, endothelial activation, adhesion molecule expression	Reduced lymphocyte apoptosis, B cell maturation, adhesion molecule expression	[30]
*IL6*	Monocytes, fibroblasts, B cells, T cells	CD4^+^ T cells	B cell proliferation, immunoglobulin production, hematopoiesis, thrombopoiesis, T cell proliferation, differentiation, cytotoxicity, acute phase response, effects on the release of monocytic cytokines	B and T cell activation, induction of cytokine responses	[31]
*IL10* (interleukin-10)	T cells, B cells, monocytes, others	CD3^+^ T cells, CD4^+^ T cells	Immune regulatory cytokine, inhibition of T cell activation, B cell differentiation, activation, and immunoglobulin production	B cell activation, (auto-) antibody production	[32, 33••, 34–36]
*IL13* (interleukin-13)	T cells, B cells, monocytes, others	CD4^+^ T cells	Closely related to IL-4 with similar function	Unclear, potentially reduced lymphocyte apoptosis, B cell maturation, adhesion molecule expression	[36]
*IL17A* (interleukin 17A)	T cells, NK cells, mast cells, neutrophils	CD3^+^ T cells, CD4^+^ T cells, effector CD4^+^ T cells	Induction of chemokines, cytokines, recruitment of neutrophils: defense against bacteria and fungi	Induction of tissue damage in SLE	[19, 37•, 38, 39]
*IL17F* (interleukin 17F)	T cells, NK cells, mast cells, neutrophils	CD3^+^ T cells, CD4^+^ T cells	Induction of chemokines, cytokines, recruitment of neutrophils: defense against bacteria and fungi	Increased CREMα-recruitment > reduced expression of IL-17F > increased IL-17A homodimers > enhanced inflammation	[16, 37•, 38, 39]
*IRF7* (interferon regulatory factor 7)	Constitutively expressed by lymphoid tissues and plasmacytoid dendritic cells; inducible in many cells and tissues	CD4^+^ T cells	Transcriptional activation of virus-inducible cellular genes, including type I interferon genes	“Interferon signature” gene; global immune activation	[29]
*ITGAL* (integrin alpha L gene, encoding for CD11A)	T cells	CD4^+^ T cells	Cellular adhesion and costimulation	Increased T cell-mediated inflammation	[40–43]
*KIR2DL4* (killer cell immunoglobulin-like receptor 2DL4, encoding for KIR)	On the surface of NK cells and some T cells	CD4^+^ T cells	Detection of virally infected cells or transformed cells	Increased expression of KIR on T cells, resulting in T cell activation	[44–46]
*Micro RNAs* (*miR98*, *miR188-3p*, *miR421*, *miR50*)	?	CD3^+^ T cells	Regulation of E3 ubiquitin-protein ligase CBL	Suppression of CBL, which downregulates T cell receptor signaling and is decreased in lupus T cells	[47]
*Micro RNA miR886*	?	Naive CD4^+^ T cells	Regulation of interferon-inducible phosphorylated RNA-dependent protein kinase	Impaired regulation of nuclear factor kappa B (NF-κB) signaling with effects on inflammation and apoptotic cell death	[48]
*MX1* (myxovirus resistance 1 gene, encoding for interferon-induced GTP-binding protein Mx1, or MxA)	Neutrophils, leukocytes	Neutrophils	GTPase, mediates resistance against RNA viruses	“Interferon signature” gene; global immune activation	[26•]
*PP2A* (serine/threonine protein phosphatase 2A)	Eukaryotic cells	CD3^+^ T cells, CD4^+^ T cells	Phosphatase with complex functions in many aspects of cell function	Increased PP2A expression in T cells from SLE patients mediates DNA demethylation through suppression of MAPK signaling pathways; induces epigenetic remodeling of the *IL17* locus	[37•, 49, 50]
*PRF1* (perforin)	CD8^+^ cytotoxic T cells and NK cells	CD4^+^ T cells	Cytolytic protein	Perforin expression in CD4^+^ T cells may contribute to T cell-induced death of monocytes/macrophages in SLE	[51, 52]
*TNFSF5* (tumor necrosis factor ligand superfamily member 5, encoding for CD40L/CD154)	Activated T cells	CD4^+^ T cells	Costimulatory molecule, B cell maturation and activation	Increased B cell costimulation and antibody production	[45, 51, 53–55]
*TNFSF7* (tumor necrosis factor ligand superfamily member 7, encoding for CD70)	Activated T cells	CD4^+^ T cells	B cell activation, IgG synthesis, T cell costimulation	Increased B cell activation, IgG synthesis, T cell costimulation	[2, 4, 56, 57]
Increased DNA methylation					
*CD8A*, *CD8B* (cluster of differentiation 8A and 8B)	CD8^+^ T cells	CD4^+^ T cells, CD8^+^ T cells, DN T cells	Co-receptor to the CD3/T cell receptor complex	Generation of CD3^+^ CD4^−^ CD8^−^ DN T cells	[14•, 58]
*IL2* (interleukin-2)	T cells	CD3^+^ T cells, CD4^+^ T cells, effector CD4^+^ T cells	Proliferation and activation of T cells	Impaired generation of regulatory T cells, reduced activation-induced cell death, and longer survival of autoreactive T cells; impaired function of cytotoxic CD8^+^ T cells, effector CD4^+^ T cell differentiation, and cytokine expression	[13, 17, 39, 59]
*FOXP3* (forkhead-box-protein P3)	T_reg_	PBMCs	Master regulator during the development and function of T_reg_	Reduced number and altered function of regulatory T cells	[60–62]
*NOTCH1* (Notch-1 trans-membrane receptor)	T cells	CD3^+^ T cells, CD4^+^ T cells	Role during T cell lineage determination, e.g., polarization of T helper cells	T cell activation, increased IL-17A expression	[18]
*NR3C1* (nuclear receptor subfamily 3 group C member 1, encoding for glucocorticoid receptor)	Multiple cells and tissues	PBMCs	Regulates development, metabolisms, and immune responses; exerts pleiotropic effects in different cells and tissues	Unknown, potentially increased immune activation	[63]

Various molecular mechanisms have been identified as contributing to altered DNA methylation in immune cells from patients with SLE (Table 2):

**Table 2:** DNA hydroxymethylation in SLE

Gene	Cellular sources	Cell type studied	Function	Effects in SLE	Ref.
3826 genes with altered hydroxymethylation	–	PBMCs	–	Immune dysregulation	[64]
*CDKN1A* (*cyclin-dependent kinase inhibitor 1A*) *increased* hydroxymethylation	Ubiquitously expressed	PBMCs	Regulation of G1 cell cycle progression, regulation of DNA replication and DNA damage repair, role during apoptosis	Unclear, potential effects on apoptosis and DNA repair	[64]
*CDKN1B* (*cyclin-dependent kinase inhibitor 1B*) *decreased* hydroxymethylation	Ubiquitously expressed	PBMCs	Regulation of G1 cell cycle progression	Unclear, probably defects in apoptosis or autophagy, resulting in increased exposure to nuclear autoantigens	[64]
*TREX1* (*3′ exonuclease TREX1*) *increased* hydroxymethylation	Ubiquitously expressed	PBMCs	DNA repair, proof reading during DNA replication; association with the SET complex, central for granzyme A-mediated cell death	Impaired exonuclease function and cytosolic DNA accumulation; reduced granzyme A-mediated cell death and survival of autoreactive cells	[64, 65]
2748 genes with increased DNA hydroxymethylation	–	CD4^+^ T cells	–	Immune dysregulation	[66]
47 genes with reduced DNA hydroxymethylation	–	CD4^+^ T cells	–	Immune dysregulation	[66]
*SOCS1* *increased* DNA hydroxymethylation		CD4^+^ T cells	Negative regulator of cytokine expression, central role in T_reg_ integrity and function	Immune dysregulation, exact mechanisms unclear	[66]
*NR2F6* (V-erbA-related protein 2 (EAR-2)) *increased* DNA hydroxymethylation	Ubiquitous expression, particularly high in liver	CD4^+^ T cells	Nuclear orphan receptor, suppression of lymphocyte activation and Th17 responses	Potentially increased autoreactive T cell activation	[66]
*IL15RA* (*IL-15 receptor alpha*) *increased* DNA hydroxymethylation	Immune cells, particularly macrophages, NK cells, and CD4^+^ and CD8^+^ T cells	CD4^+^ T cells	Promotion of T cell proliferation and activation	Potentially increased autoreactive T cell proliferation and activation	[66]

### Altered DNMT Expression and Activity in SLE

In several studies of T cells from patients with SLE, reduced expression of DNMT1 and DNMT3a was demonstrated. However, conflicting reports exist suggesting no differences in the expression of DNMTs in lymphocytes from SLE patients vs. healthy controls. Reported differences may be due to variable disease activity of SLE patients included in the studies, variable ethnicities, and the possibility that mRNA expression may not reflect protein expression or activity of DNMTs in the studied populations [67–69]. Furthermore, DNMT recruitment and activity are largely signal-, target-, and tissue-specific and can be directed by transcription factors (see below), and mitogen-activated protein (MAP) kinases [2–4, 37•, 49, 50].

### Mitogen Activated Protein Kinases

Altered methylation of genomic DNA in lymphocytes from SLE patients was linked to uncontrolled activation of mitogen activated protein kinases (MAPK). Impaired activation of protein kinase C (PKC)δ results in reduced activation of extracellular signal-regulated kinases (ERK) and impaired DNMT1 activity, subsequently contributing to reduced DNA methylation and increased expression of the costimulatory molecules CD11A, CD70, CD40L, the pro-inflammatory effector cytokine IL-17A, and several interferon-regulated genes [3, 4, 70•, 71]. Another mechanism contributing to reduced DNMT1 expression is the increased expression of protein phosphatase 2A (PP2A), which suppresses ERK signaling and DNMT1 activity [49, 50].

### Growth-Arrest and DNA Damage Inducible Protein45α

The growth-arrest and DNA damage inducible protein (GADD)45α is expressed at increased levels in T cells from SLE patients. In a highly complex manner, it induces DNA demethylation through the interaction with activation-induced deaminase (AID) and MBD4, involving 5-methyl-cytosine-deaminase and G:T mismatch-specific thymine glyosylase [4, 72]. GADD45α interacts with the regulatory protein high mobility group box (HMGB)1, which in turn functionally interacts with MeCP2, a protein that is centrally involved in the recognition of methylated DNA, directing DNA methylation. Together, these mechanisms result in gradual DNA demethylation of *ITGAL* (encoding for CD11A) and *TNFSF7* (encoding for CD70) in T cells from patients with SLE [73].

### Dysregulated Transcription Factor Networks

During the differentiation of the lymphocyte population, transcription factors instruct epigenetic remodeling, thereby defining the phenotype of cells and tissues. Altered transcription factor networks are a hallmark of SLE T cells [2–4, 65]. Expression of the transcription factor cAMP responsive element (CREM)α is increased in T cells from SLE patients and reflects disease activity [74]. CREMα instructs epigenetic remodeling of SLE-associated genes through its interaction with DNMT3a, contributing to the generation of effector T cells in SLE [2–4, 13, 14•, 15–19, 74].

### TET Proteins and DNA Hydroxymethylation

More recently, DNA hydroxymethylation was considered an epigenetic event [75] and to be involved in the pathophysiology of autoimmune/inflammatory disease, including SLE [76]. DNA hydroxymethylation can act as an intermediary in the process of active DNA demethylation [77, 78••, 79, 80]. In various cells and tissues, positive correlation between gene expression and DNA hydroxymethylation has been demonstrated. DNA hydroxymethylation is the result of oxidation of methylated cytosines within CpG dinucleotides by the hydroxytransferase ten eleven translocation (TET) family proteins [77, 80–82, 83••]. DNA hydroxymethylation results in reduced affinity of DNA to MBDs and increased transcription factor binding. Thus, DNA hydroxymethylation is currently considered a permissive epigenetic mark, promoting gene expression [64, 84, 85]. In agreement with these reports, TET family mRNA expression positively correlates with increased DNA hydroxymethylation in SLE. However, DNA hydroxymethylation patterns are complex and incompletely understood with areas of increased and reduced hydroxymethylation [3, 66, 76] (Table 2).

## Non-coding-RNAs

Transcription of non-coding RNAs from either intronic or intergenic regions of the genome is potentially important for the regulation of gene expression. Though highly interesting and promising in the search for molecular mechanisms contributing to altered gene expression in autoimmune/inflammatory conditions, our understanding of the physiological as well as the pathophysiological role in gene expression is very limited. Furthermore, the question of whether non-coding RNAs should be considered an epigenetic event or not remains somewhat controversial. Based on their general heritability, the involvement gene regulation without affecting the underlying DNA sequence, non-coding RNAs fulfill the criteria of epigenetic mechanisms of gene regulation. Non-coding RNA expression occurs at the interface between the transcription of genes, chromatin remodeling, and the translation of messenger RNA into protein products, regulating approximately 30% of human genes [86]. This may partially be achieved by providing an “open” chromatin conformation by ongoing transcriptional activity. During this, non-coding transcripts mediate interactions between core promoters and enhancers, which may be located far apart, sometimes even on different chromosomes [87]. However, the function of non-coding RNAs is not limited to providing an “open” chromatin conformation. Non-coding RNAs can be processed by the nuclear ribonuclease Drosha and the cytoplasmic Dicer enzyme. Resulting micro RNAs (miRNAs) are usually 21–23 base-pair spanning processed transcripts that can interfere with gene expression through duplex formation with target genes or transcripts, usually at the 3′ untranslated region (3′ UTR) [3, 4, 87–92], resulting in transcriptional repression, mRNA cleavage, or translational arrest [3, 89–92]. Non-coding RNA expression can be either the result or the cause of other epigenetic alterations, and several connections between non-coding RNA expression and DNA methylation have been established: miRNA29 and miRNA143 influence DNA methylation through the regulation of DNMT3a and DNMT3b [93–96]. In cancer, miRNA126 was linked with reduced MAPK activity and subsequently reduced DNMT1 expression in T cells from SLE patients [3, 97].

## Histone Modifications

In addition to DNA methylation and the effects of non-coding RNAs discussed above, post-translational modifications of histone proteins regulate gene expression on the epigenetic level. In the nucleus of eukaryotic cells, histone proteins aggregate to octamers with two copies of each H2A, H2B, H3, and H4. Histone octamers form complexes with genomic DNA (147 base-pairs). These complexes are referred to as nucleosomes. Histone proteins undergo post-translational modifications at amino acid termini which serve three-dimensional arrangement of nucleosomes, controlling the accessibility to transcriptional factors and finally gene expression [2–4]. Important histone modifications include acetylation, citrullination, phosphorylation, and methylation. Activating histone modifications that confer chromatin “opening” include histone H3 lysine 18 acetylation (H3K18ac). Conversely, histone H3 lysine 9 (H3K9me3) and/or lysine (H3K27me3) trimethylation mediate chromatin condensation and transcriptional silencing. A number of enzymes and multiprotein complexes have been suggested to mediate specific epigenetic marks [2–6], which include lysine acetyltransferases (HATs), HDACs, lysine methyltransferases (KMTs), and lysine demethylases (KDMs) [98].

Epigenetic marks are highly specific and determine the phenotype and function of cells and tissues. Disturbed histone marks centrally contribute to the pathophysiology of autoimmune/inflammatory disorders, including SLE. Both histone acetylation and histone H3K9 methylation are decreased in CD4^+^ T cells from SLE patients [99] (Table 3). However, histone modifications are complex and very incompletely understood [2, 4]. Alterations to the histone code have extensively been studied in cytokine genes. T cells from SLE patients exhibit permissive modifications to histone proteins at the *IL17* gene cluster (increased H3K18ac and reduced levels of H3K27me3) contributing to uncontrolled expression of pro-inflammatory IL-17A [16, 19]. Conversely, the *IL2* gene undergoes epigenetic silencing in T cells from SLE patients. Reported histone modifications along the *IL2* gene are repressive with impaired histone acetylation and increased methylation. Together, these histone modifications contribute to the effector phenotype of T cells from SLE patients [13, 16, 17, 19] (Table 3). In concert with the involvement in disrupted DNA methylation, the transcription factor CREMα plays a role in these events through its interaction with HDAC1 and the histone methyltransferase G9a [14•, 17].

**Table 3:** Histone modifications in SLE

Gene	Modification	Cell type studied	Function	Effects in SLE	Ref.
*CD8A*, *CD8B* (cluster of differentiation 8A and 8B)	H3K18 deacetylation, H3K27 trimethylation in DN T cells •Epigenetic silencing	CD4^+^ T cells, CD8^+^ T cells, DN T cells	Co-receptor to the CD3/T cell receptor complex	Generation of CD3^+^ CD4^−^ CD8^−^ DN T cells	[14•, 58]
*ITGAL* (integrin alpha L gene, encoding for CD11A)	Reduced H3K27 trimethylation through histone demethylase JMJD3	CD4^+^ T cells	Cellular adhesion and costimulation	Increased T cell-mediated inflammation	[100]
*IL2* (interleukin-2)	H3K18 deacetylation, H3K27 trimethylation •Epigenetic silencing	CD3^+^ T cells, CD4^+^ T cells, effector CD4^+^ T cells	Proliferation and activation of T cells	Impaired generation of regulatory T cells, reduced activation-induced cell death, and longer survival of autoreactive T cells; impaired function of cytotoxic CD8^+^ T cells, effector CD4^+^ T cell differentiation, and cytokine expression	[13, 17, 39, 59]
*IL10* (interleukin-10)	H3K18 acetylation •Increased gene expression	CD3^+^ T cells, CD4^+^ T cells	Immune regulatory cytokine, inhibition of T cell activation, B cell differentiation, activation, and immunoglobulin production	B cell activation, (auto-) antibody production	[32, 33••, 34, 35]
*IL17A* (interleukin 17A)	H3K18 acetylation •Increased gene expression	CD3^+^ T cells, CD4^+^ T cells, effector CD4^+^T cells	Induction of chemokines, cytokines, recruitment of neutrophils: defense against bacteria and fungi	Induction of tissue damage in SLE	[19, 37•, 38, 39]
*TNF* (tumor necrosis factor alpha)	H3 acetylation •Increased gene expression	Monocytes	Monocyte activation, cytokine, and prostaglandin production, priming of mononuclear cells, apoptosis, and oxidative burst, induction of endothelial cell adhesion molecules and cytokine release and T cell apoptosis	Increased monocyte maturation and pro-inflammatory cytokine expression	[101]

Another cytokine undergoing significant dysregulation in lymphocytes from SLE patients is IL-10. While generally considered an immune regulatory or anti-inflammatory cytokine, IL-10 also has pro-inflammatory effects. It contributes to B cell proliferation, differentiation, and activation as well as the induction of antibody production and immunoglobulin class switch [32, 87, 102]. Enhanced IL-10 expression in SLE was linked with high disease activity. Indeed, a small cohort of treatment resistant SLE patients responded to IL-10 blockade with antibodies [103]. We demonstrated that, in T cells from SLE patients, *IL10* undergoes epigenetic remodeling through DNA demethylation and histone acetylation [33••] (Table 3). The transcription factor Stat3 that is over-activated in T cells from patients with SLE is centrally involved through its interactions with the histone acetyltransferase p300 [33••].

## Demographic Factors and Environment

As many other autoimmune/inflammatory conditions, SLE is characterized by female predominance (f:m = 9–10:1). Because prevalence of SLE in pre-pubertal children is about equal in girls and boys, hormones appear central in the pathophysiology of SLE. Estrogens have been most widely studied in SLE and indeed are involved in T cell subset differentiation and distribution through epigenetic remodeling [2, 4, 20, 56, 104–107]. Estrogen receptor signaling enhances the expression of the transcription factor CREMα, which greatly contributes to the generation of effector CD4^+^ T cell and DN T cells in SLE [2, 4, 13, 14•, 16, 17, 19, 74].

Furthermore, the presence of a second X chromosome may contribute to the increased prevalence of SLE in women. Most X-linked genes are not gender-specific and exhibit equal expression rates. A complex epigenetic event referred to as “X chromosome inactivation” is responsible for stable gene expression. X inactivation involves the aforementioned epigenetic events, DNA methylation, histone modifications, and miRNA expression. Several X-linked genes contribute to the pathophysiology of SLE [2, 4, 108]. Reduced DNA methylation of *CD40L* contributes to female predominance of SLE [53, 54, 109, 110]. Furthermore, women who lack one X chromosome (Turner syndrome: 45, X0) exhibit lower incidences of SLE. Conversely, individuals with an additional X chromosome (Klinefelter’s syndrome: 47, XXY) are at an increased risk for the development of SLE [2, 4, 111, 112].

Elderly men exhibit greater SLE incidences when compared to elderly women. With increasing age, epigenetic events appear to accumulate and impact gene expression even more than genetic predisposition [51]. A possible explanation is reduced DNMT1 activity in the elderly [2, 4, 57, 65]. One result of cumulative DNA demethylation may be the generation and accumulation of “senescent” T cells that are characterized by reduced CD28 expression, shortened telomeres, and increased expression of SLE-associated genes [2, 30, 44, 113].

Several routinely used medications cause epigenetic alterations that represent a well-accepted environmental trigger for inflammation [2–4]. Furthermore, modifications to DNA and/or histone proteins depend on substrates derived from diet or products of intermediary metabolism. Through methionine adenosyltransferase (MAT), a redox-sensitive enzyme in the S-adenosyl methionine (SAM) cycle, SAM derives from adenosine triphosphate (ATP) and methionine [114]. Thus, the availability of B vitamins and methionine directly regulate SAM generation. Global DNA methylation is reduced in SLE patients and in the elderly, suggesting alterations to the SAM cycle and/or DNMT activity as likely contributors to DNA demethylation [2, 56, 115]. Particularly in individuals with reduced DNMT1 activity, sufficient nutritional intake of SAM may be essential to prevent autoimmune reactions [115•]. Hydralazine (used to treat hypertension) inhibits the activity of proteinkinase Cδ, resulting in impaired ERK kinase activation and subsequently altered activity of DNMT1 [70•, 71, 116]. Through these mechanisms, hydralazine mediates DNA demethylation and lupus-like phenotypes in predisposed individuals.

Lastly, sunlight exposure triggers flares in SLE patients [117, 118]. Indeed, UV exposure results in reduced DNMT1 mRNA expression and reduced DNA methylation in T cells from SLE patients [118]. This may be due to the induction of GADD45α through UV light promoting DNA demethylation and altered gene expression [4, 72, 119].

## The Epigenome as Therapeutic Target

In contrast to the situation in cancer, therapeutic approaches “officially labelled” as epigenetic treatment are not available for SLE. However, several currently available therapeutic agents modify the epigenome. In general, epigenetic treatment strategies in autoimmune/inflammatory conditions are limited by untargeted effects and the fact that epigenetic patterns in SLE are highly complex. Thus, untargeted approaches may cause severe adverse events. In the presence of relatively well-established alternative (though often toxic) treatment options, epigenetic approaches are currently considered unethical and risky [2–4].

Available therapeutic regimens in SLE include antimalaria medication (chloroquine and hydroxy-chloroquine), corticosteroids, and immune-modulating agents (methotrexate, mycophenolate mofetil, and cyclophosphamide) [3].

### DNA Methylation

Methotrexate reduces DNMT1 activity through the depletion of SAM, the substrate of DNMTs during DNA methylation [3, 120, 121]. Cyclophosphamide treatment in systemic vasculitis increases DNA methylation through the induction of DNMT1 activity [3, 122]. Thus, epigenetic effects of methotrexate and cyclophosphamide may explain effectiveness in SLE [3]. DNA methylation can furthermore be altered by 5′-azacytidine (Vidaza) or 5′-aza-2′-deoxycytidine (Decitabine), cytosine analogues that integrate into DNA during cell division and prevent DNA methylation [123].

Though several therapeutic interventions influence DNA hydroxymethylation, no targeted approaches are available to correct altered DNA hydroxymethylation. Treatment of RA patients with methotrexate reduced DNA hyroxymethylation [76, 124], and inhibition of TAT family proteins by the IDH1 inhibitors AGI-5198 or HMS-101 reduce DNA hydroxymethylation and exert in vitro effects on tumor cell proliferation. Thus, TET inhibition may prove useful in autoimmune/inflammatory conditions, including SLE [124–126].

### Micro RNAs

Several miRNAs are targetable by small molecules. Some of them have already made their way into preclinical studies in infectious hepatitis C and cancers [127–129]. However, no data exist yet concerning miRNA blockade in autoimmune/inflammatory disorders.

### Histone Modifications

Though histone modifying enzymes have not been “directly” targeted in SLE yet, several therapeutic regimens alter histone modifications [3]. Several currently available drugs inhibit HDACs, including the antiepilepic valproic acid, vorinostat (Zolinza), and romidepsin (Istodax), both used in T cell lymphoma. Increased histone acetylation was suggested to be beneficial in SLE, since global histone acetylation is reduced in T cells from SLE patients [4]. Indeed, HDAC inhibition with suberoylanilide hydroxamic acid (SAHA) or trichostatin A (TSA) results in clinical improvement of disease in lupus-prone mice [130, 131]. Conversely, application of the “HDAC inhibitor” valproic acid in epilepsy patients sometimes results in lupus-like symptoms [132]. Thus, currently available epigenetic treatment may be limited by global effects that may cause adverse reactions that outweigh potential benefits.

Mycophenolate mofetil influences the histone code, while not affecting DNA methylation [133]. Histone methyltransferase G9a mediates methylation at Histone H3K9 and H3K27 termini, both repressive epigenetic modifications. G9a inhibitors have been developed and are currently under investigation in preclinical cancer studies [134].

### Future Interventions

Disrupted transcription factor networks are a hallmark of T lymphocytes from SLE patients [1]. As mentioned above, T cells from SLE patients are characterized by increased expression and activation of CREMα and increased Stat3 activation. Both transcription factors centrally contribute to the inflammatory phenotype of SLE through the induction of epigenetic remodeling [2, 3, 13, 14•, 33]. Thus, blocking transcription factor expression or activation appears promising in the search for target-directed treatment options in SLE and other autoimmune/inflammatory diseases. To date, blockade of Stat transcription factors signaling is already achievable through Janus kinase (JAK) inhibitors. However, JAK inhibition is currently not part of standard treatment protocols, and its role in future approaches remains to be determined [135].

## Conclusions

Immune cells from patients with the systemic autoimmune disease SLE are characterized by dysregulated gene expression profiles. A significant proportion is caused by epigenetic alterations. Over the past years, a number of molecular mechanisms have been linked with epigenetic dysregulation. Altered epigenetic marks may therefore be the “missing link” between genetic predisposition and disease expression in SLE but also in other autoimmune/inflammatory disorders. Provided the sometimes highly specific epigenetic patterns in lymphocytes from SLE patients, epigenetic events hold potential in the search for targets for individualized therapeutic interventions and disease biomarkers. However, additional studies are warranted focusing on target-directed alterations to the epigenome in autoimmune/inflammatory disorders.
